# Optimization of SgRNA expression with RNA pol III regulatory elements in *Anopheles stephensi*

**DOI:** 10.1038/s41598-025-98557-0

**Published:** 2025-04-18

**Authors:** Estela Gonzalez, Michelle A. E. Anderson, Joshua X. D. Ang, Katherine Nevard, Lewis Shackleford, Mireia Larrosa-Godall, Philip T. Leftwich, Luke Alphey

**Affiliations:** 1 Arthropod Genetics, The Pirbright Institute, GU24 0NF Pirbright, UK; 2 Animal and Plant Health Agency, Woodham Lane, KT15 3NB Addlestone, Surrey UK; 3 Department of Biology, University of York, Wentworth Way, YO10 5DD York, UK; 4 School of Biological Sciences, University of East Anglia, Norwich Research Park, NR4 7TJ Norwich, UK

**Keywords:** CRISPR/Cas9, Gene drive, Mosquito, Malaria, Genetic engineering, Synthetic biology, Gene expression

## Abstract

*Anopheles stephensi*, a major Asian malaria vector, is invading Africa and has been implicated in recent outbreaks of urban malaria. Control of this species is key to eliminating malaria in Africa. Genetic control strategies, and CRISPR/Cas9-based gene drives are emerging as promising species-specific, environmentally friendly, scalable, affordable methods for pest control. To implement these strategies, a key parameter to optimize for high efficiency is the spatiotemporal control of Cas9 and the gRNA. Here, we assessed the ability of four RNA Pol III promoters to bias the inheritance of a gene drive element inserted into the *cd* gene of *An. stephensi*. We determined the homing efficiency and examined eye phenotype as a proxy for non-homologous end joining (NHEJ) events in somatic tissue. We found all four promoters to be active, with mean inheritance rates up to 99.8%. We found a strong effect of the Cas9-bearing grandparent (grandparent genotype), likely due to maternally deposited Cas9.

## Introduction

*Anopheles stephensi* mosquitoes have been involved in the transmission of urban malaria in South Asia and the Arabian Peninsula for many decades^1^. The recent invasion of this species into Africa threatens malaria control efforts by adding a new vector, especially in urban environments where endemic *Anopheles* mosquitoes were absent^2–4^. In a few years, *An. stephensi* has spread from East to West Africa and has been implicated in several urban malaria outbreaks^5–8^. This is a particular concern both because more people are at risk, and because there are indications that this species is responsible for the spread of drug and diagnosis resistant strains of *Plasmodium falciparum*^5^. Control of this species has become a priority, with the WHO releasing an initiative to stop the spread of *An. stephensi* in Africa in 2023^3,9–11^.

Malaria control has reached a plateau in which traditional methods seem less effective, mainly due to the emergence and spread of insecticide resistance. Hence, new mosquito control strategies are needed and are being actively developed. Genetic control methods have emerged as a potentially powerful strategy since they are species-specific, environmentally friendly methods for controlling pests. More specifically, gene drives have emerged as potential genetic biocontrol tools for controlling pest populations threatening global health^12–15^. These aim to bias the inheritance of a genetic element such that, under suitable circumstances, it can increase in frequency in a population even if it imposes some fitness cost on individual carriers. For CRISPR/Cas9 based gene drives to be efficient, several parameters need to be optimised, most importantly the spatiotemporal expression of Cas9 and sgRNA. Development of a toolbox for engineering this vector is urgently needed, with most current investigations instead focusing on other important disease vectors such as *Aedes aegypti* and *An. gambiae*^15–19^.

To help fill this toolbox, this study focuses on sgRNA expression, using four different RNA Pol III promoters with Cas9 under the endogenous regulation of *zero population growth* (*zpg*) and targeting *cardinal* (*cd*), a component of the ommochrome pathway. Ommochrome is the major eye pigment in mosquitoes and *cd* null individuals display a distinctive, pink-eyed phenotype^19^. The use of split Cas9 and sgRNA components, each integrated into a specific locus, allows the effect of the Pol III promoter to be investigated accounting for issues due to position effect, a well-known phenomenon with transgenics where chromosome location can alter the expression pattern of promoters within a transgene^20,21^. The use of the *cd* gene as a target also allows us to separately measure overall cleavage rates, combining homing (inheritance of the *cd* integrated, sgRNA expressing element) and NHEJ events (pink-eyed phenotype), making this a useful tool for optimization of both the Cas9 and sgRNA components enabling the determination of which is the limiting factor in the cells at the time at which homing occurs.

The results of this study show that all four RNA Pol III promoters tested were active in both male and female germlines of *An. stephensi*, inducing mean inheritance bias up to 99.8%. We also reveal a strong effect of the sex of the Cas9-bearing grandparent, likely due to maternal deposition of Cas9. Such maternally-derived Cas9 can then cleave alleles in the developing embryo which are repaired by NHEJ resulting in indels which are resistant to further cleavage. Strategies for gene drives to circumvent this resistance include multiplexing approaches which typically use multiple sgRNAs to target a gene, thus alleles with an indel at one target still have several target sites available for homing^16,22–24^. This study provides multiple functional Pol III promoters which greatly facilitates this type of resistance-management strategy.

## Results

For each promoter we generated an insertion into *cd* only differing by the RNA Pol III promoter and terminator sequences used to express the sgRNA. The *cd* gene encodes a heme peroxidase, an enzyme in the ommochrome pathway^19^. Like the *kmo* gene, another gene in this pathway which has been widely used for transgenesis studies, homozygous null mutants for these genes display depigmentation in the mosquito eyes which allows for screening of homing events (fluorescent marker) and non-homologous end joining (NHEJ) events, in the case of *cd* giving a pink-eyed phenotype. Deposition or somatic expression of Cas9 may result in somatic NHEJ events leading to a mosaic-eyed phenotype. Here, we selected the *cd* gene, since previous studies showed deleterious effects on the *kmo* null mutant^25^.

**Fig. 1:**
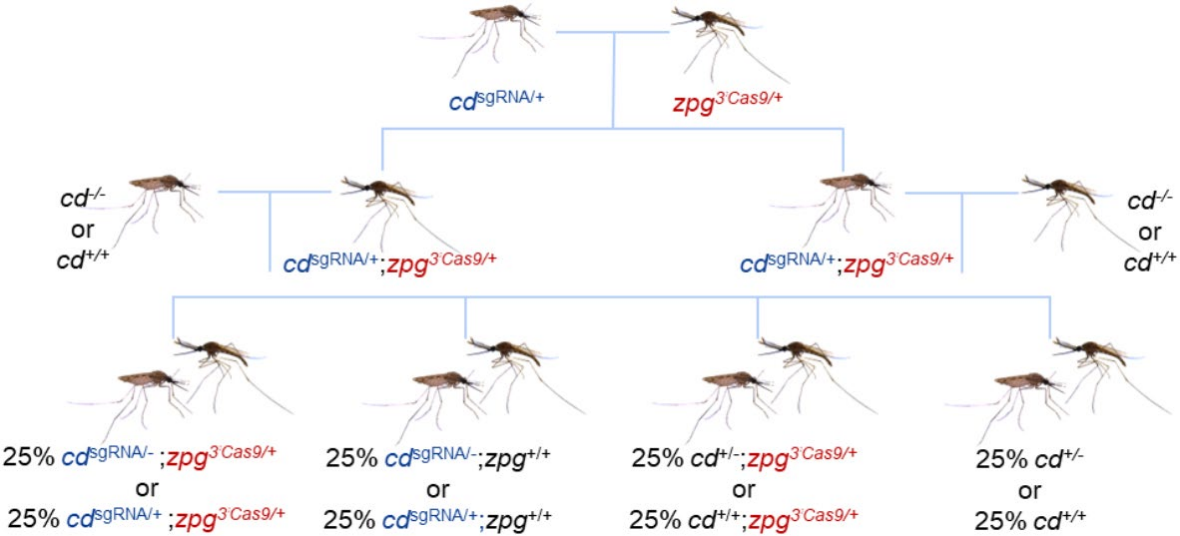
Crossing scheme for inheritance assessments. *cd*^sgRNA^ females were crossed to *zpg*^3’Cas9^ males. The transheterozygous F_1_ were then crossed either to WT or to *cd*^−/−^ of the opposite sex. The F_2_ generation was screened for the presence of the fluorescent markers as well as eye phenotype.

To study the effect of the promoter, each of the lines (*cd*^sgRNA^) was mated to a transgenic line expressing Cas9 under the endogenous control of *zpg* (*zpg*^3’Cas9^). Transheterozygous F_1_ individuals were then crossed to wild type strain (*cd*^+/+^) to measure the inheritance bias and effects of deposited Cas9 or to a *cd*^−/−^ line which allows assessment of the overall cleavage rate (Fig. 1). This allows the detection of differences in homing and/or cutting efficiencies by screening the F_2_ offspring for transgene inheritance and eye phenotype.

### Pol III promoter affects inheritance rate

**Fig. 2:**
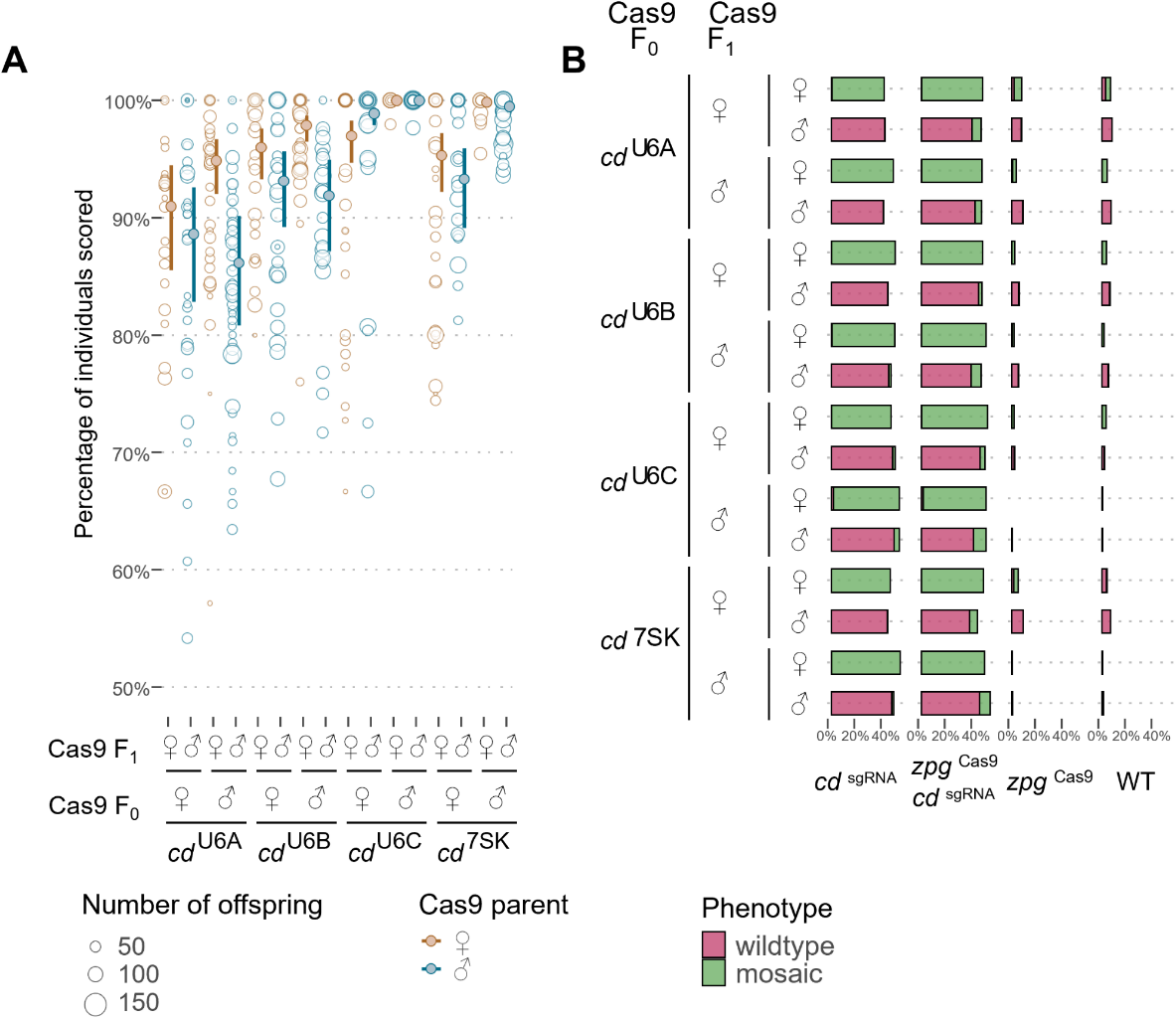
Pol III promoters bias the inheritance of a gene drive element in the *An. stephensi* germline. (**A**) Open circles represent inheritance rates from a single parent, size indicates the number of offspring per female. Darker, filled points and error bars are estimated means and 95% confidence intervals. (**B**) Eye phenotypes were determined to be wild type (pink bars) or mosaic/pink eyes (green bars) and are presented as the proportion of the total F_2_ separated by genotype.

The four promoters assessed were found to be active in both the male and female germlines of *An. stephensi* (Data file D1). More specifically, those crosses using the U6-C and 7SK promoters had the highest overall rates of inheritance (mean [95% CI]; U6-C, 99.8% [99.6–99.9]; 7SK, 98.7% [98.15–99.1]). Moreover, these two lines also showed an effect depending on the sex of the Cas9-bearing grandparent (U6-C, z = 6.1, *p* < 0.001; 7SK, z = 6.36, *p* < 0.001). There was also a significant but reduced effect of the Cas9-bearing parent in U6-C (z = 2.47, *p* = 0.014). In both instances the highest rates of homing, with means approaching 100%, were observed in the offspring of male Cas9-bearing grandparents (Fig. 2A, Table S2).

Examination of the eye phenotype showed that mosaicism was dependent on the sex of the Cas9-bearing parent. The results reveal that 100% of the F_1_ progeny coming from a Cas9-bearing female and carrying the *cd*^sgRNA^ element displayed mosaic eyes. Furthermore, almost all offspring of the F_1_ transheterozygous females also showed mosaic eyes, whether they inherited the transgenes or not, while very few mosaics were found in the offspring of the F_1_ transheterozygous males. This indicates that across all lines, the observed rates of mosaicism could be predicted almost exclusively by the parental genotype (LRT: χ21 = 30915, *P* < 0.001, Partial R2 = 0.96; Fig. 2B) and supports the conclusion that maternal deposition of Cas9 results in mosaic progeny.

## Cleavage rate mediates inheritance bias

In order to determine overall cleavage rates, we also crossed *zpg*^3’Cas9^;*cd*^sgRNA^ transheterozygotes to *cd*^−/−^. Progeny of this cross inherit one null *cd* allele from their *cd*^−/−^ parent and either the *cd*^sgRNA^ transgene or the putatively WT (or cut and repaired by NHEJ/homing) allele from the transheterozygote parent. Mosaicism in those progeny not carrying the *cd*^sgRNA^ indicates that the WT allele was cut somatically (not in all cells). Complete pink eyes would indicate a germline cut repaired by NHEJ into a non-functional allele. Careful observation of the rates of each allows the estimation of the cleavage and NHEJ rates. This is necessarily an approximation, since a very early or high level of embryonic cutting could conceivably give fully pink eyes.

**Fig. 3:**
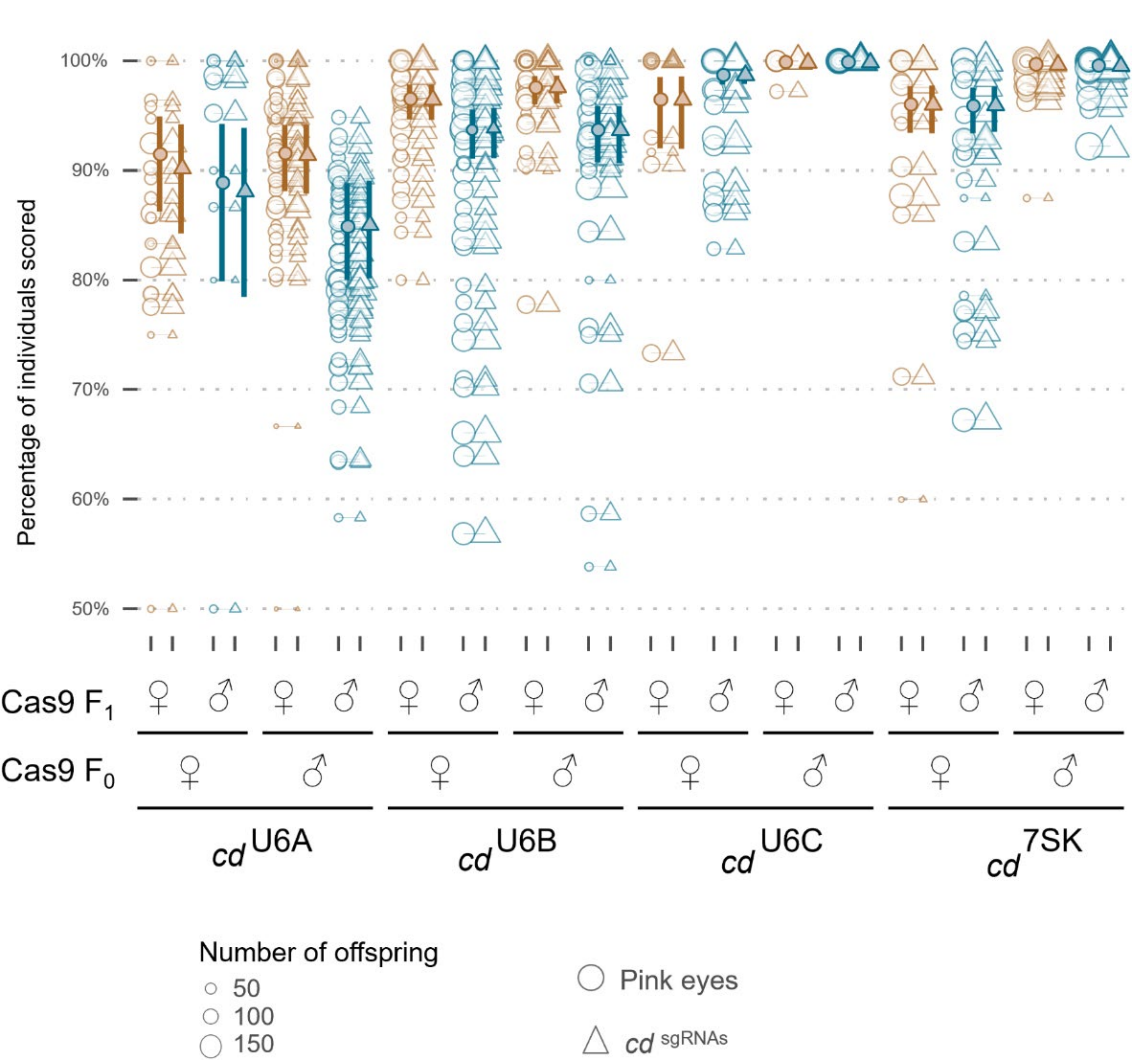
Homing and cleavage rates across four independent transgenic lines crossed to pink eyed strain. Open circles represent individual inheritance rates from a single parent, size of the circle indicates the number of offspring. Darker, filled points and error bars are estimated means and 95% confidence intervals. Triangles represent the proportion of the F_2_ which inherit the *cd*^sgRNA^ element. Circles represent the proportion of the F_2_ which have a pink eyed phenotype indicating an indel in *cd*.

We observed inheritance rates similar to those found in the cross to WT from the section above (Data file D1). Again, a very strong effect from the sex of the Cas9-bearing grandparent was detected (U6-C, z = 4.45, *p* < 0.001; 7SK, z = 6.44, *p* < 0.001). However, in this set of crosses, we observed a significant but reduced effect of the Cas9-bearing parent in U6-C only (z = 2.14, *p* = 0.03) (Fig. 3, Table S2). Interestingly, we found that the cleavage rate was equivalent to the inheritance of the drive element for all the promoters except U6A, and for U6C it was nearly 100%. This is likely an indication of expression levels varying with the different promoters. In addition, the high correlation between overall cleavage rate and drive inheritance may also indicate that the expression pattern and/or timing of U6B, U6C and 7SK overlaps with the *zpg* derived expression of Cas9 during a time window which is most favorable for homology-directed repair (HDR), with very few cleavage events resulting in NHEJ. It may be that for homing gene drives there are optimal pairs/sets of RNA Pol II and RNA Pol III promoters which express at the same time and lead to high drive efficiency, and these may need to be optimised for different species or different drive systems.

## Discussion

Our data are consistent with previous observations of maternally deposited Cas9 resulting in somatic cleavage, and very little paternal deposition^26–29^.This indicates that these Pol III promoters were active in early stages of embryonic development, and therefore even if any of them are not deposited maternally, maternal deposition of Cas9 alone can still result in resistance allele formation. To mitigate this effect, methods to further restrict expression of Cas9 to the germline should be investigated. This has been the most problematic issue from the beginning of the development of gene drive strains in *An. stephensi*. For example, Gantz et al. utilized the *vasa* promoter to express Cas9 in an autonomous gene drive. They reported near 100% inheritance of the drive through the paternal lineage, however this was significantly reduced through the maternal lineage due to maternal deposition of Cas9 and creation of resistant alleles.

Our results pave the way for future gene drive designs with multiple sgRNAs expressed by multiple RNA Pol III promoters to mitigate resistance. In these systems, multiple sgRNAs are expressed all targeting the same gene, such that alleles which are resistant to one sgRNA may still be cleaved by another sgRNA and the drive is able to spread. Although methods for expressing several sgRNAs from a single transcript such as using tRNAs, ribozymes or Csy4 have been reported in cell culture models, many of these are yet to be validated in mosquitoes and multiple, functional RNA Pol III promoters present a simple alternative. While previous gene drive systems in *An. stephensi* have mostly utilized a single RNA Pol III promoter, U6A^26,28^, we have identified three previously uncharacterized highly functional RNA Pol III promoter sequences in *An. stephensi* which are more active than the U6A promoter commonly used for sgRNA expression. These promoters could all generate up to 100% cutting/biased inheritance in the male and female germlines, although U6C and 7SK outperformed the others with mean inheritance rates near 100%.

Interestingly, we have found U6A to be the least active promoter in conjunction with our *zpg* expressed Cas9, suggesting that there is an expression level and/or spatiotemporal difference between the different promoters. To our knowledge, the only other study which has shown this was Li et al.^18^. In their hands, wGDe-U6b when assessed with exu-Cas9, exhibited the highest homing rate compared to the other promoters (U6a, U6b, and U6d) while U6d, which in a previous experiment was shown to be functional, seemed to be inactive when inserted into the target locus. This should not be a surprise, despite being a critical component for mRNA splicing^30^, there are multiple copies present in the genome and it is likely that some loci may have developed ‘tunability’ to cater to the requirements of different cell types. Further insight into the expression pattern of RNA Pol III promoters, could aid in the optimisation of the homing process. This type of investigation would be challenging, at least for the U6 genes as the RNA they express is identical. Robust characterization of sgRNA expression presents significant technical challenges as these are very short, highly structured RNAs. Little is known as to the ‘correct’ spatiotemporal window for homing to occur and extensive research into this could significantly advance the field. In the meantime, having several options of RNA Pol III promoters to express sgRNAs could aid in this investigation. These multiple, functional promoters for sgRNA expression should be useful for many gene drive applications in this species.

## Materials and methods

### Plasmids

Four insertions into *cd* expressing the same sgRNA_384 (cut site corresponds to roughly amino acid 384 of *cardinal*: GCAGCAACTTAATCAAGCCA*CGG*, PAM site italicised) from different RNA pol III promoters were generated. The sgRNA target was identified using CHOPCHOP^31^ and a high-ranking target site within the desired region, with no variation annotated in Vectorbase^32,33^ and no off-targets was selected. Three endogenous U6 RNA promoters (ASTE015697-U6A, ASTE015521-U6B, ASTE015587-U6C) as well as the 7SK (ASTE015331) RNA promoter were identified by BLAST using the U6 or 7SK RNA as a query. A CLUSTAL alignment of the promoter sequences used was generated and putative conserved proximal promoter elements (TATA box, PSE) identified (Figure S1). The construct design consists of approximately 1.5 kb of homology 5’ and 3’ of the cut site for the sgRNA to introduce a fluorescent marker (Hr5/IE1-AmCyan-K10) and the sgRNA expression cassette (Pol III promoter–sgRNA–terminator) into the genome by HDR.

The Cas9 is expressed by utilizing the endogenous *zpg* locus (ASTE011088) as an integral element. Plasmid AGG1590 (*zpg*^3’Cas9^) was used to integrate a ubiquitin monomer followed by the Cas9 ORF and marker expression cassette (3xP3-mCherry-SV40) immediately following the *zpg* coding sequence just upstream of the stop codon by HDR.

Full plasmid sequences are available in NCBI (accession numbers: PQ005639-PQ005643).

### Mosquito rearing

All experiments performed for this study were approved by the Biological Agents and Genetic Modification Safety Committee of The Pirbright Institute and are in accordance with all relevant regulations. *An. stephensi* SDA-500 strain (WT) were maintained at 28 °C, 75–85% relative humidity and 14:10 light/dark cycle^34^. Larvae were reared as 200 larvae per 500 ml RO water in 6 L trays and fed Seramicron (Sera) as early instars and ExtraSelect (Su-Bridge Pet Supplies Ltd) as later instars. Adults were provided with 10% sucrose *ad libitum* and blood-fed defibrinated horse blood (TCS Bioscience) using a Hemotek membrane feeder (Hemotek Ltd) with parafilm (Bemis).

## Generation of mosquito Transgenic lines

Microinjections on WT embryos were performed 1–2 h post oviposition, as previously described^34^. Injection mixes contained 300ng/µl Cas9 (PNABio), 50ng/µl in vitro transcribed sgRNA^37^ and 800ng/µl HDR donor plasmid.

Surviving G_0_ mosquitoes were pool crossed to WT as 20 G_0_ males:100 WT females, and 20 G_0_ females:20 WT males. Females were blood-fed and eggs were collected and hatched. G_1_ larvae were screened under a Leica MZ165C fluorescence microscope for the presence of the fluorescent marker (Table S1). Positive G_1_ individuals were mated to WT to continue the line and the insertions confirmed by PCR and Sanger sequencing (Figure S2).

*cd*^*−/−*^ mosquitoes used for the cleavage assay crosses were generated by sibling crossing the non-fluorescent F_2_ progeny from a *cd*^U6A^;*zpg*^3’Cas9^ homing cross and screening their progeny for eye phenotype. The line was established with two mutations, confirmed by PCR and Sanger sequencing, but after several generations only one mutation remained; this was a 3 bp deletion which resulted in amino acids Q383 and A384 changing to P (Figure S3).

## Crosses for drive and cleavage assessment

Males and females, heterozygous for the *cd*^sgRNA^ element, were crossed reciprocally with heterozygous mosquitoes of the *zpg*^3’Cas9^ line (F_0_). Their progeny (F_1_) were screened for fluorescence and eye phenotype. For the homing assessment, at least 20 transheterozygous *cd*^sgRNA^;*zpg*^3’Cas9^ mosquitoes of each sex were crossed to WT mosquitoes of the opposite sex, blood-fed and females separated into individual cups and allowed to lay eggs. The F_2_ were hatched, reared and screened for fluorescence and eye phenotype. For the cleavage assessment, at least 20 transheterozygous *cd*^sgRNA^;*zpg*^3’Cas9^ of each sex were crossed to *cd*^−/−^ of the opposite sex, also allowed to lay individually, and their F_2_ progeny scored for transgene inheritance indicated by fluorescence and cleavage indicated by eye phenotype.

### Statistical analysis

Analyses were carried out using R version 4.4.0^35^. Estimated means and 95% confidence intervals were calculated by a Generalized Linear Mixed Model, with a binomial (‘logit’ link) error distribution fitted using the glmmTMB package^38^. Where applicable, initial parameters included transgenic line, sex of the Cas9-bearing G_1_ parent and G_0_ grandparent; individual replicates were included as a random effect. The model was summarized with ‘emmeans’^39^ and model residuals were checked for violations of assumptions using the ‘DHARMa’ package^40^. Figures were generated with ggplot2^41^. Where applicable a partial McFadden’s R^2^ was calculated by comparing deviance of full and partial models. Scripts and raw data can be found at Github (https://github.com/Philip-Leftwich/Optimization-sgRNA-Anopheles-stephensi).

## Electronic supplementary material

Below is the link to the electronic supplementary material.

Supplementary Material 1

Supplementary Material 2

## Data Availability

All data generated for this study is available in the main manuscript and supplementary files. Full plasmid sequences are available from NCBI accession numbers: PQ005639-PQ005643.

## References

[1] Ishtiaq, F., Swain, S. & Kumar, S. S. *Anopheles stephensi* (Asian malaria Mosquito). *Trends Parasitol.* **37**, 571–572 (2021). 33865712 10.1016/j.pt.2021.03.009

[2] Sinka, M. E. et al. A new malaria vector in Africa: Predicting the expansion range of *Anopheles stephensi* and identifying the urban populations at risk. *Proc. Natl. Acad. Sci.* 117, 24900–24908 (2020). 10.1073/pnas.2003976117 PMC7547157 32929020

[3] Faulde, M. K., Rueda, L. M. & Khaireh, B. A. First record of the Asian malaria vector *Anopheles stephensi* and its possible role in the resurgence of malaria in Djibouti, Horn of Africa. *Acta Trop.* **139**, 39–43 (2014). 25004439 10.1016/j.actatropica.2014.06.016

[4] Zhou, G., Zhong, D., Yewhalaw, D. & Yan, G. *Anopheles stephensi* ecology and control in Africa. *Trends Parasitol.* **40**, 102–105 (2024). 38142196 10.1016/j.pt.2023.11.011 PMC11849806

[5] Emiru, T. et al. Evidence for a role of *Anopheles stephensi* in the spread of drug- and diagnosis-resistant malaria in Africa. *Nat. Med.* **29**, 3203–3211 (2023). 37884028 10.1038/s41591-023-02641-9 PMC10719088

[6] Hawaria, D. et al. First report of *Anopheles stephensi* from Southern Ethiopia. *Malar. J.* **22**, 373 (2023). 38066610 10.1186/s12936-023-04813-x PMC10704791

[7] Al-Eryani, S. M. et al. Public health impact of the spread of *Anopheles stephensi* in the WHO Eastern mediterranean region countries in Horn of Africa and Yemen: need for integrated vector surveillance and control. *Malar. J.* **22**, 187 (2023). 37337209 10.1186/s12936-023-04545-y PMC10278259

[8] Santi, V. P. et al. Role of *Anopheles stephensi* mosquitoes in malaria outbreak, Djibouti, 2019 - 27, number 6—June 2021 - Emerging infectious diseases journal - CDC. 10.3201/eid2706.204557 10.3201/eid2706.204557 PMC8153885 34013869

[9] WHO initiative to stop the spread of. *Anopheles stephensi* in Africa, 2023 update. https://www.who.int/publications/i/item/WHO-UCN-GMP-2022.06

[10] Whittaker, C. et al. Seasonal dynamics of *Anopheles stephensi* and its implications for mosquito detection and emergent malaria control in the Horn of Africa. *Proc. Natl. Acad. Sci.* 120, e2216142120 (2023). 10.1073/pnas.2216142120 PMC9974477 36791102

[11] Ahmed, A., Abubakr, M., Ali, Y., Siddig, E. E. & Mohamed, N. S. Vector control strategy for *Anopheles stephensi* in Africa. *Lancet Microbe*. **3**, e403 (2022). 35659899 10.1016/S2666-5247(22)00039-8

[12] Alphey, L. S., Crisanti, A., Randazzo, F., (Fil) & Akbari, O. S. Standardizing the definition of gene drive. *Proc. Natl. Acad. Sci.* **117**, 30864–30867 (2020). 33208534 10.1073/pnas.2020417117 PMC7733814

[13] Burt, A. Site-specific selfish genes as tools for the control and genetic engineering of natural populations. *Proc. R Soc. Lond. B Biol. Sci.* **270**, 921–928 (2003). 10.1098/rspb.2002.2319 PMC1691325 12803906

[14] Deredec, A., Burt, A. & Godfray, H. C. J. The population genetics of using homing endonuclease genes in vector and pest management. *Genetics* **179**, 2013–2026 (2008). 18660532 10.1534/genetics.108.089037 PMC2516076

[15] Hammond, A. et al. Gene-drive suppression of mosquito populations in large cages as a Bridge between lab and field. *Nat. Commun.* **12**, 4589 (2021). 34321476 10.1038/s41467-021-24790-6 PMC8319305

[16] Anderson, M. A. E. et al. A multiplexed, confinable CRISPR/Cas9 gene drive can propagate in caged *Aedes aegypti* populations. *Nat. Commun.* **15**, 729 (2024). 38272895 10.1038/s41467-024-44956-2 PMC10810878

[17] Kyrou, K. et al. A CRISPR–Cas9 gene drive targeting doublesex causes complete population suppression in caged *Anopheles gambiae* mosquitoes. *Nat. Biotechnol.* **36**, 1062–1066 (2018). 30247490 10.1038/nbt.4245 PMC6871539

[18] Li, M. et al. Germline Cas9 expression yields highly efficient genome engineering in a major worldwide disease vector, Aedes aegypti. *Proc. Natl. Acad. Sci.* **114**, E10540–E10549 (2017). 29138316 10.1073/pnas.1711538114 PMC5724270

[19] Carballar-Lejarazú, R. et al. Next-generation gene drive for population modification of the malaria vector mosquito, Anopheles gambiae. *Proc. Natl. Acad. Sci.* **117**, 22805–22814 (2020). 32839345 10.1073/pnas.2010214117 PMC7502704

[20] Markstein, M., Pitsouli, C., Villalta, C., Celniker, S. E. & Perrimon, N. Exploiting position effects and the Gypsy retrovirus insulator to engineer precisely expressed transgenes. *Nat. Genet.* **40**, 476–483 (2008). 18311141 10.1038/ng.101 PMC2330261

[21] Roberts, J. A. et al. Targeted transgene integration overcomes variability of position effects in zebrafish. *Dev. Camb. Engl.* **141**, 715–724 (2014). 10.1242/dev.100347 PMC3899822 24449846

[22] Edgington, M. P., Harvey-Samuel, T. & Alphey, L. Population-level multiplexing: A promising strategy to manage the evolution of resistance against gene drives targeting a neutral locus. *Evol. Appl.* **13**, 1939–1948 (2020). 32908596 10.1111/eva.12945 PMC7463328

[23] Yang, E. et al. A homing suppression gene drive with multiplexed gRNAs maintains high drive conversion efficiency and avoids functional resistance alleles. *G3 GenesGenomesGenetics* 12, jkac081 (2022). 10.1093/g3journal/jkac081 PMC9157102 35394026

[24] Hou, S. et al. A homing rescue gene drive with multiplexed gRNAs reaches high frequency in cage populations but generates functional resistance. *J. Genet. Genomics*. **51**, 836–843 (2024). 38599514 10.1016/j.jgg.2024.04.001

[25] Bottino-Rojas, V. et al. Beyond the eye: kynurenine pathway impairment causes midgut homeostasis dysfunction and survival and reproductive costs in blood-feeding mosquitoes. *Insect Biochem. Mol. Biol.* **142**, 103720 (2022). 34999199 10.1016/j.ibmb.2022.103720 PMC11055609

[26] Adolfi, A. et al. Efficient population modification gene-drive rescue system in the malaria mosquito *Anopheles stephensi*. *Nat. Commun.* **11**, 5553 (2020). 33144570 10.1038/s41467-020-19426-0 PMC7609566

[27] Verkuijl, S. A. N., Anderson, M. A. E., Alphey, L. & Bonsall, M. B. Daisy-chain gene drives: the role of low cut-rate, resistance mutations, and maternal deposition. *PLOS Genet.* **18**, e1010370 (2022). 36121880 10.1371/journal.pgen.1010370 PMC9521892

[28] Gantz, V. et al. Highly efficient Cas9-mediated gene drive for population modification of the malaria vector mosquito Anopheles stephensi. *Proc. Natl. Acad. Sci.* **112**, E6736–E6743 (2015). 26598698 10.1073/pnas.1521077112 PMC4679060

[29] Pham, T. B. et al. Experimental population modification of the malaria vector mosquito, *Anopheles stephensi*. *PLOS Genet.* **15**, e1008440 (2019). 31856182 10.1371/journal.pgen.1008440 PMC6922335

[30] Das, G., Henning, D., Wright, D. & Reddy, R. Upstream regulatory elements are necessary and sufficient for transcription of a U6 RNA gene by RNA polymerase III. *EMBO J.* **7**, 503–512 (1988). 3366121 10.1002/j.1460-2075.1988.tb02838.x PMC454347

[31] Labun, K. et al. CHOPCHOP v3: expanding the CRISPR web toolbox beyond genome editing. *Nucleic Acids Res.* **47**, W171–W174 (2019). 31106371 10.1093/nar/gkz365 PMC6602426

[32] Giraldo-Calderón, G. I. et al. VectorBase: an updated bioinformatics resource for invertebrate vectors and other organisms related with human diseases. *Nucleic Acids Res.* **43**, D707–D713 (2015). 25510499 10.1093/nar/gku1117 PMC4383932

[33] Giraldo-Calderón, G. I. et al. VectorBase.org updates: bioinformatic resources for invertebrate vectors of human pathogens and related organisms. *Curr. Opin. Insect Sci.* **50**, 100860 (2022). 34864248 10.1016/j.cois.2021.11.008 PMC9133010

[34] Southworth, J. et al. Expanding the transgene expression toolbox of the malaria vector *Anopheles stephensi*. *Insect Mol. Biol.* **34**, 104–110 (2025). 39129057 10.1111/imb.12953 PMC11705503

[35] Core Team, R. R: A language and environment for statistical computing. R Foundation for Statistical Computing. Available from: https://www.r-project.org/

[36] Catteruccia, F., Benton, J. P. & Crisanti, A. An Anopheles Transgenic sexing strain for vector control. *Nat. Biotechnol.* **23**, 1414–1417 (2005). 16244659 10.1038/nbt1152

[37] Bassett, A. & Liu, J. L. CRISPR/Cas9 mediated genome engineering in *Drosophila*. *Methods* **69**, 128–136 (2014). 24576617 10.1016/j.ymeth.2014.02.019

[38] Brooks, M. GlmmTMB balances speed and flexibility among packages for Zero-inflated generalized linear mixed modeling. *R J.* **9**, 378 (2017).

[39] Lenth, R. V. & emmeans Estimated Marginal Means, aka Least-Squares Means. (2023).

[40] Hartig, F. DHARMa: residual diagnostics for hierarchical (multi-level/mixed) regression models. (2022).

[41] Wickham, H. *Ggplot2* (Springer International Publishing, 2016). 10.1007/978-3-319-24277-4

