# Supplementary material for: Optimization of SgRNA expression with RNA pol III regulatory elements in Anopheles stephensi
Source: Sci Rep. 2025 Apr 18;15:13408. doi: 10.1038/s41598-025-98557-0 (PMC12008393; doi:10.1038/s41598-025-98557-0)
Supplement: Supplementary file 2 — Supplementary Material 2 [file 41598_2025_98557_MOESM2_ESM.docx]

**Supplementary information file**

**Optimization of sgRNA expression with RNA Pol III regulatory elements in *Anopheles stephensi***

Estela Gonzalez^1,2†^, Michelle A. E. Anderson^1,3†^, Joshua X. D. Ang^1,3^, Katherine Nevard^1^, Lewis Shackleford^1,3^, Mireia Larrosa-Godall^1^, Philip T. Leftwich^4^, Luke Alphey^1,3^*

^†^these authors contributed equally

*Email address: luke.alphey@york.ac.uk

^1^Arthropod Genetics, The Pirbright Institute, Pirbright GU24 0NF, U.K.

^2^Animal and Plant Health Agency, Woodham Lane, Addlestone, Surrey, KT15 3NB

^3^Department of Biology, University of York, Wentworth Way, York, YO10 5DD

^4^School of Biological Sciences, University of East Anglia, Norwich, Norfolk NR4 7TJ, U.K.

**Table S1. Summary of injections**

| Line | HDR donor plasmid | Embryos injected | G_0_ survivors | G_1_ screened (positives) |
| --- | --- | --- | --- | --- |
| *cd*^U6A^ | AGG1626 | 1,359 | 93 (6.8%) | 9,322 (90) |
| *cd*^U6B^ | AGG1757 | 2,165 | 12 (0.6%) | 728 (112) |
| *cd*^U6C^ | AGG1758 | 1,720 | 56 (3.2%) | 7,147 (107) |
| *cd*^7SK^ | AGG1759 | 1,124 | 17 (1.5%) | 2,648 (51) |
| *zpg*^3’Cas9^ | AGG1590 | 2,164 | 96 (4.4%) | 7,607 (6) |

**Table S2. Model summaries for homing against WT, *cd*^-/-^ and for cleavage rates against *cd*^-/-^.**

|  | **Homing against WT** | **Homing against cd^-/-^** | **Cutting against cd^-/-^** |
| --- | --- | --- | --- |
| *Predictors* | *Log-Odds* | *Log-Odds* | *Log-Odds* |
| Intercept | 2.28 ^***^ (1.75 – 2.81) | 2.41 ^***^ (1.87 – 2.96) | 2.26 ^***^ (1.71 – 2.82) |
| Cas9 parent (Male) | -0.21 (-0.83 – 0.42) | -0.39 (-1.08 – 0.30) | -0.32 (-1.02 – 0.39) |
| Cas9 grandparent (Male) | 0.66 ^*^ (0.04 – 1.27) | -0.03 (-0.66 – 0.59) | 0.10 (-0.53 – 0.74) |
| *cd*^U6B^ | 0.87 ^*^ (0.17 – 1.58) | 0.97 ^**^ (0.28 – 1.65) | 1.12 ^**^ (0.42 – 1.81) |
| *cd*^U6C^ | 1.18 ^**^ (0.41 – 1.96) | 0.93 (-0.11 – 1.97) | 1.09 ^*^ (0.03 – 2.15) |
| *cd*^7SK^ | 0.72 (-0.00 – 1.45) | 0.83 ^*^ (0.08 – 1.57) | 0.98 ^*^ (0.22 – 1.74) |
| Cas9 parent:  grandparent | -0.92 ^**^ (-1.53 – -0.30) | -0.25 (-0.86 – 0.37) | -0.29 (-0.92 – 0.33) |
| Parent: *cd*^U6B^ | -0.32 (-1.08 – 0.44) | -0.31 (-1.04 – 0.42) | -0.37 (-1.11 – 0.38) |
| Parent: *cd*^U6C^ | 1.22 ^*^ (0.22 – 2.23) | 1.45 ^*^ (0.21 – 2.69) | 1.40 ^*^ (0.14 – 2.66) |
| Parent: *cd*^7SK^ | -0.15 (-1.00 – 0.70) | 0.30 (-0.52 – 1.13) | 0.25 (-0.59 – 1.09) |
| Grandparent: *cd*^U6B^ | 0.04 (-0.72 – 0.80) | 0.34 (-0.44 – 1.12) | 0.23 (-0.56 – 1.03) |
| Grandparent: *cd*^U6C^ | 4.29 ^***^ (2.91 – 5.67) | 4.89 ^***^ (2.73 – 7.06) | 4.78 ^***^ (2.61 – 6.95) |
| Grandparent: *cd*^7SK^ | 2.82 ^***^ (1.99 – 3.66) | 2.82 ^***^ (1.95 – 3.69) | 2.72 ^***^ (1.84 – 3.60) |
| **Random Effects** | | | |
| σ^2^ | 5.03 | 4.70 | 4.77 |
| τ_00_ | 1.74 _id:Cas9_parent:Cas9_grandparent:Line_ | 1.41 _id:Cas9_parent:Cas9_grandparent:Line_ | 1.48 _id:Cas9_parent:Cas9_grandparent:Line_ |
|  | 0.00 _Cas9_parent:Cas9_grandparent:Line_ | 0.00 _Cas9_parent:Cas9_grandparent:Line_ | 0.00 _Cas9_parent:Cas9_grandparent:Line_ |
|  | 0.00 _Cas9_grandparent:Line_ | 0.00 _Cas9_grandparent:Line_ | 0.00 _Cas9_grandparent:Line_ |
|  | 0.00 _Line_ | 0.00 _Line_ | 0.00 _Line_ |
| N | 649 _id_ | 549 _id_ | 549 _id_ |
|  | 2 _Cas9_parent_ | 2 _Cas9_parent_ | 2 _Cas9_parent_ |
|  | 2 _Cas9_grandparent_ | 2 _Cas9_grandparent_ | 2 _Cas9_grandparent_ |
|  | 5 _Line_ | 5 _Line_ | 5 _Line_ |
| Observations | 649 | 549 | 549 |
| Marginal R^2^ / Conditional R^2^ | 0.476 / NA | 0.437 / NA | 0.437 / NA |
| ** p<0.05   ** p<0.01   *** p<0.001* | | | |

ASTE015331_As 7SK TTTCAATCAATGAATGATTTCATTCAT-------------CAGCAACAAAACAATCTCCA 60

ASTE015521_As U6B ---------ATGGACGATAGAATAAGACCTCCTTAAAATACTTTATTCAAGCTATTGCTT 60

ASTE015587_As U6C --------------------TCTACGCCCTGCTC----CCCACCAGGCAGACACAGGCAG 60

ASTE015697_As U6A -CTCAA-CTGCCGACGAGTTGCTCGGGTTTCGATGAGTTCCAGCATAC--GCGCATGCTG 60

* * * * *

ASTE015331_As 7SK TCACTTTCTTCATTGAAGATTTTACAAGAATC---------------------------A 120

ASTE015521_As U6B TCGAT-TCTTCATTGAGATCTAATCTCAAATTTGTTTATTTAGAAATAAAACA------G 120

ASTE015587_As U6C GCAAGGTCTATTCCGAAAGGAGCCGAATAATAATCAACAATACAATTAAAATAGACAGCC 120

ASTE015697_As U6A CTGAGTGCTTCTTGGTCGCATATACACTCATGCCCTTTTTTCCTTGT------------G 120

** * **

ASTE015331_As 7SK ACTGTGCGTACCAGTATTCCTAAAC-----AAAGAATGCTTTCCCGAACAAAAGAATGGA 180

ASTE015521_As U6B CGAATATTGTGCTGTAATAACTCTTTGGTAATCTTAGGCTTCCAGAAATGATAGAATCCT 180

ASTE015587_As U6C ATGGTAATTTGTTGATTGAACA-TG-------TTTACGCATTACAAAAAGAAACGCTCAA 180

ASTE015697_As U6A AAGATATTTTTTTCTATTCAAAATTTCAA---------ATTTGAAGGCAATTTTCGTGCT 180

* * *

ASTE015331_As 7SK TGAGAGCAATGAGAGACTACGCATCGAGCATCGACTCTCCGCGACTGTTGTAAGGTTCCG 240

ASTE015521_As U6B TAAGACTGCAAG------A-ATATACAAGTTT-----TTAAGAAAGGAAAACAGCTATAT 240

ASTE015587_As U6C AA-CCCCAAAGC---ACCA-ACACCAACAACT-----GTAACAACGGTTTTATTGTGCGT 240

ASTE015697_As U6A TATGTTTAATAA---TCTA-TAAAAGAAAATT-----TAAAACTAAAAACTCATGTACAG 240

* * * *

ASTE015331_As 7SK GCGTTCGGCGAGCGGAGCTAAAAATAGATCTCAATGGCAG----GCAGCAGAAAAG---- 300

ASTE015521_As U6B CAAATCAGAAAAC---AACAACAGCTCTTTTCCGTTCAATTCTACCTGTACGATGG---C 300

ASTE015587_As U6C TTGTTCAAC-CAG---CACAATCGATTTTCTCGCCTCGCTAACACATGCGGACGCACGAC 300

ASTE015697_As U6A CAGTAGATGACGC---TTTCACCGTTCTTCTCACTTCAATTCTACCTGCACAATGG---C 300

* * ** *

ASTE015331_As 7SK CGATGCGTCGCGTGAACGCATGCGCGACATCGCAGCAGCATAGCCGCGTAGCATA----A 360

ASTE015521_As U6B -------TTAAGGACGAGCTG---C-----AACTGAAACGGAACCTTACAACAGCCACGC 360

ASTE015587_As U6C TGCGGTGTGATGCGTGCGCTTGAAG-----GGTTAAGACGGAACCTTACAAAGT-----C 360

ASTE015697_As U6A -------TGGTGGAAGAGCTA---T-----AATTGAAGCAGAACCTTATAACAGTCACGC 360

* * ** * * ** *

ASTE015331_As 7SK GAGCGACCGAGGGAGGGGGTAGCAAATGCTT**ATCCATCGCTAGA**GCTAGGTTGGG-CTGG 420

ASTE015521_As U6B AGAACGAGCAAGGACTTTGTTTT--------**ATCCATCGCTAGA**ACTAAAACGAAAGGTC 420

ASTE015587_As U6C AGCTGCTTGTCGTGTGCGATGGTACGGAGTT**ATGCATCGCTAGA**AGTAAAACGGA-CAAG 420

ASTE015697_As U6A AGAAGGATCAAGAGCTTTGTGAATATCCTTTATC**CATCGCTAGA**ACTAAAACGGA-TGAC 420

* * ** ********** ** *

ASTE015331_As 7SK ATGCGG**TATATATA**GCGAACGGCGGCACACCGTTCCCT 458

ASTE015521_As U6B AGATTG**TATATATA**CCAACCTATTTTCCCCCTTCCTT- 458

ASTE015587_As U6C CGTCGG**TATATATA**CAGTGCGCGATTGCTCCTTACTT- 458

ASTE015697_As U6A AGACGGG**ATATATA**CCAACCCATTTGCCCCCTCCCTT- 458

* ******* * * ** *

**Figure S1. CLUSTAL alignment of the genomic regions of U6A, U6B, U6C and 7SK**. The promoter sequences used in this study. Proximal Sequence Element (PSE) and TATA box in bold.


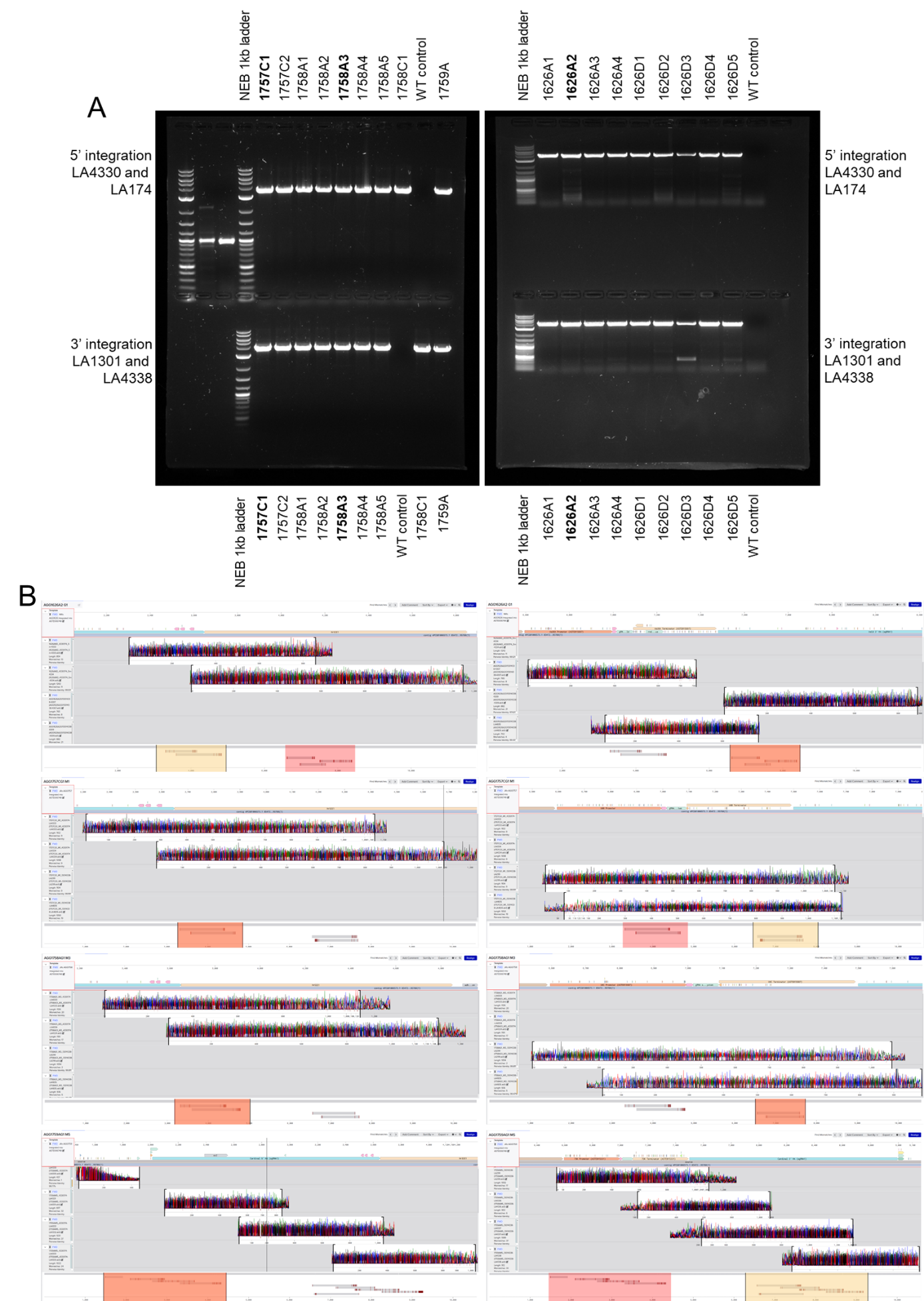


**Figure S2. Sequence confirmation of cd insertions.** Primers LA4330: CGCAGCCGAAACGTGTTCAACATTC and LA174 AGGATGTCGAAGGAGAAGGCCAGG were used to amplify from the genome into the 5’ end of the transgene and primers LA1301 TGGCCTTCTCCTTCGACATCCTGT and LA4338 AGTACATACATTCTCAACCGAAGGCGC were used to amplify from the 3’end of the transgene into the genome. Gel images (A) and screenshots from alignments performed in Benchling (<https://www.benchling.com/>) for the G_1_ individuals selected for each insertion (B). In the alignment the homology arms are shown in blue, transgene components shown in shades of orange.


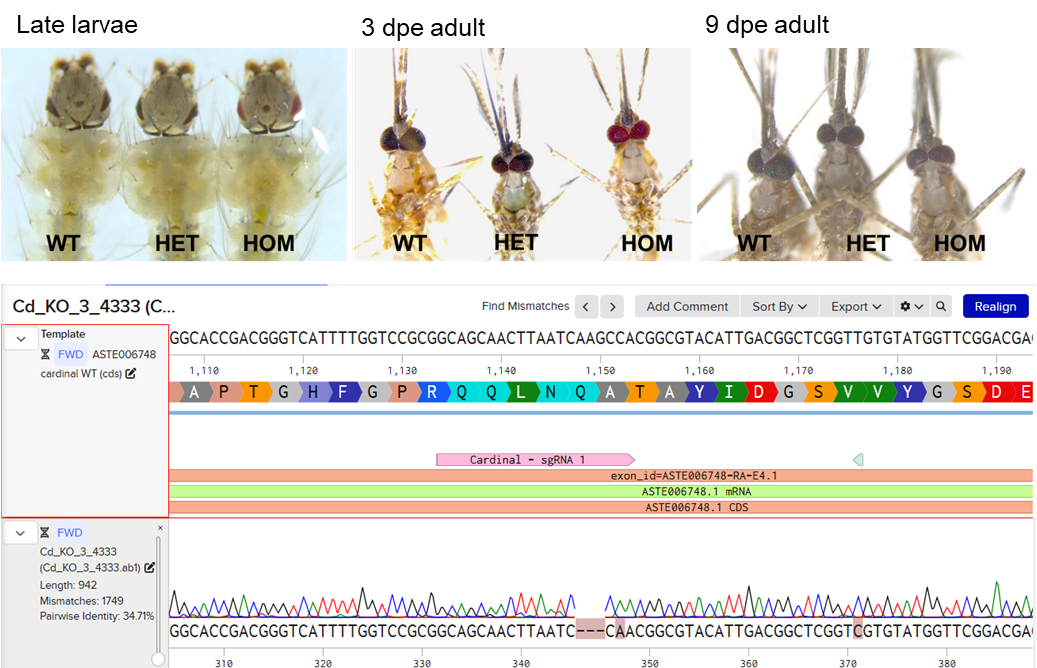


**Figure S3. Cd phenotype.** From left to right the eye phenotype of wild-type (cd^+/+^), heterozygous (cd^+/-^) and homozygous knock-out (cd^-/-^) larvae, 3 days post eclosion (dpe), and 9 dpe adult mosquitoes. Images taken on a Leica MZ165FC with a D3000 camera. WT = wild-type, HET = heterozygous, HOM = homozygous. Underneath, the Sanger sequencing results for the *cd^-/-^* line.
